# Sciatic Herpes Zoster Suspected of Lumbar Disc Herniation: An Infrequent Case Report and Literature Review

**DOI:** 10.3389/fsurg.2021.663740

**Published:** 2021-05-07

**Authors:** Fei-Long Wei, Tian Li, Yang Song, Lin-Ya Bai, Yifang Yuan, Chengpei Zhou, Jixian Qian, Xiaodong Yan

**Affiliations:** ^1^Department of Orthopeadics, Tangdu Hospital, Fourth Military Medical University, Xi'an, China; ^2^School of Basic Medicine, Fourth Military Medical University, Xi'an, China; ^3^Tangdu Hospital, Fourth Military Medical University, Xi'an, China

**Keywords:** sciatic herpes zoster, lumbar disc herniation, diagnosis, pain, VAS (analog visual scale)

## Abstract

**Background:** The symptoms of sciatic herpes zoster are sometimes difficult to distinguish from sciatica caused by lumbar disc herniation. We describe a case of suspected lumbar disc herniation with sciatic herpes zoster to reduce the rate of misdiagnosis.

**Case Report:** A 55-year old man, male, developed low back pain after carrying heavy items 20 years ago. Characteristics of symptoms: 1. Symptoms were aggravated in the upright lumbar forward flexion position; 2. The VAS (leg) score was 8–9 points and the VSA (lumbar) score was 0 point; 3. It can be relieved when rested in the supine position; 4. It came on intermittently with radiation pain in the right lower limb. There were several attacks every year. One month ago, there was radiating pain in the right lower limb. The pain was from the back of the right hip, behind the thigh, in lateral crural region, to the back of the foot. And Symptoms worsened for 10 days. The VAS score was 8 points. Pain could not be relieved by rest or changing posture. There was no back pain, no lower limbs, weak walking, no claudication and other symptoms. Analgesics and neurotrophic drugs are ineffective. After the application of antiviral drugs, the radiation pain in the right lower extremity was significantly relieved.

**Conclusion:** We describe this case in detail and discuss how to make an authentic diagnosis, with a concomitant literature review.

## BACKGROUND

Herpes zoster (HZ), often referred to as shingles or zona, is a kind of viral disease characterized by painful rashes with blisters in a limited area on one side of the body, usually in the form of streaks. Initial infection with varicella zoster virus (VZV) can cause acute illness (chickenpox), usually in children and young adults. HZ is a reactivation of the latent varicella zoster virus infection, which can lead to a large incidence, especially in the elderly and patients with impaired immune function (1). The identified risk factors for Hertz include oldness and immunosuppressive drugs (2–4).

Although the lower extremity sciatic herpes zoster is rare clinically, its symptoms and signs are very similar to those of lumbar disc herniation. Especially for the patients with a history of lumbar disc herniation and imaging of corresponding segments with nerve compression, it is easy to be confused (5). Here we report a case a case of suspected lumbar disc herniation with sciatic herpes zoster to reduce the rate of misdiagnosis.

## Case Presentation

A 55-year-old male who was admitted to our hospital in 2019 developed lower back pain after carrying heavy items 20 years ago. Characteristics of symptoms: 1. Symptoms were aggravated in the upright lumbar forward flexion position; 2. The VAS (leg) score was 8–9 points and the VSA (lumbar) score was 0 point; 3. The pain can be relieved when rested in the supine position; 4. It came on intermittently with radiation pain in the right lower limb. There were several attacks of the symptoms every year. One month ago, there was radiation pain in the right lower limb. The pain was from the back of the right hip, behind the thigh, along the lateral crural region, and to the dorsum of foot. And Symptoms worsened for 10 days. The VAS (leg) score was 8–9 points. Pain is not relieved by rest or changing posture. There was no lower back pain, weak walking, no intermittent claudication and other symptoms. Analgesics and neurotrophic drugs are almost ineffective.

### Physical Examination

There was no abnormalities in the appearance of the spine and limbs. L4/5, L5/S1 spinous process gap tenderness is positive; limb sensation, muscle strength and muscle tension were all normal. Knee reflex: left/right = + +/−; ankle reflex: left/right = ++/+; pathological sign (−). Lasegue sign: left/right = −/+ (60°); Bragard sign: left/right = −/−; Femoral nerve tension test: left/right = -/-; Piriformis strain test: left/right = −/−.

### Laboratory Tests

There were no obvious abnormalities in laboratory tests such as blood routine, electrolyte, liver and kidney function, C-reactive protein, and erythrocyte sedimentation rate.

**Figure 1:**
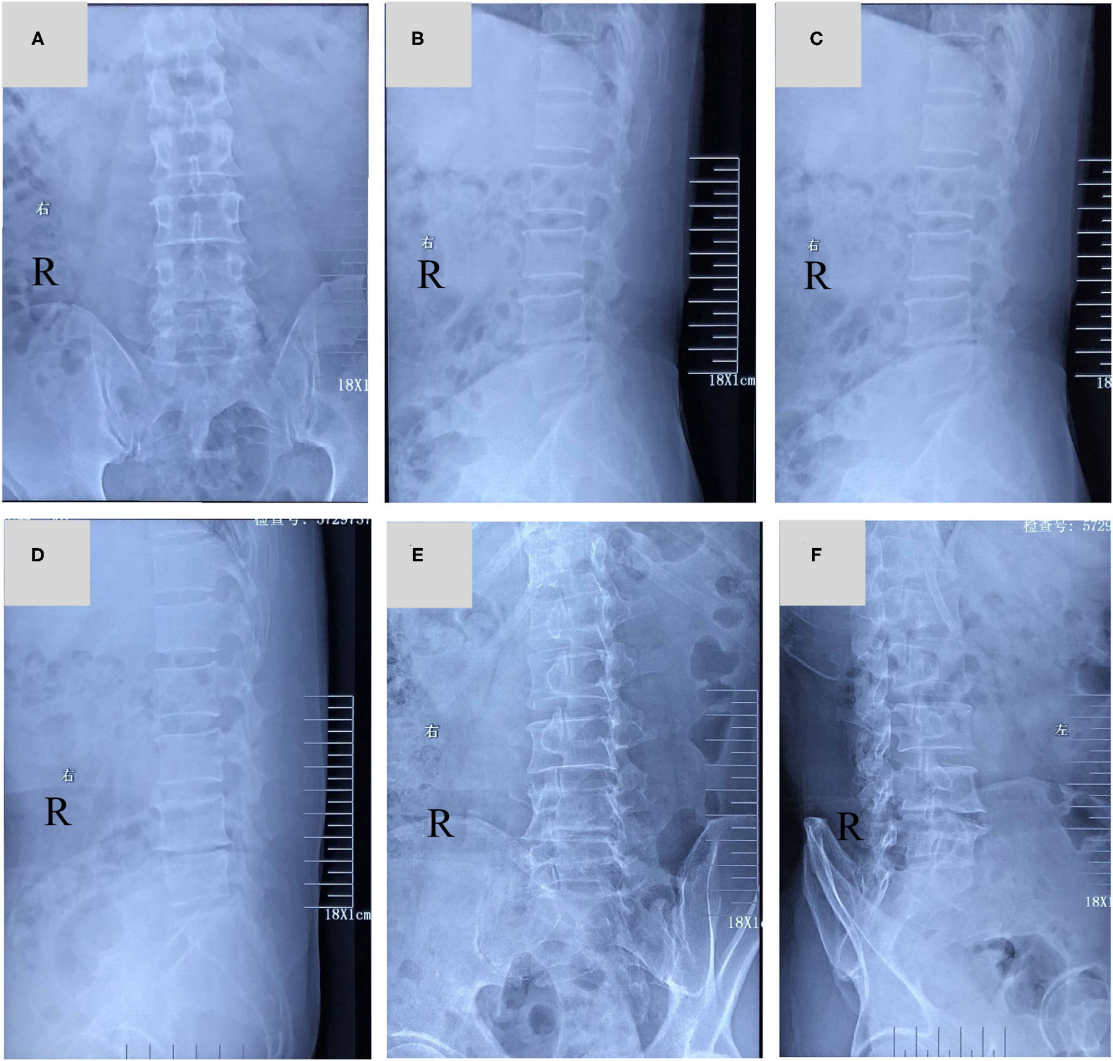
**(A–F)** lumbar vertebrae X-ray in 6 position: X-ray films showed that the L4/5 intervertebral height was obviously collapsed.

**Figure 2:**
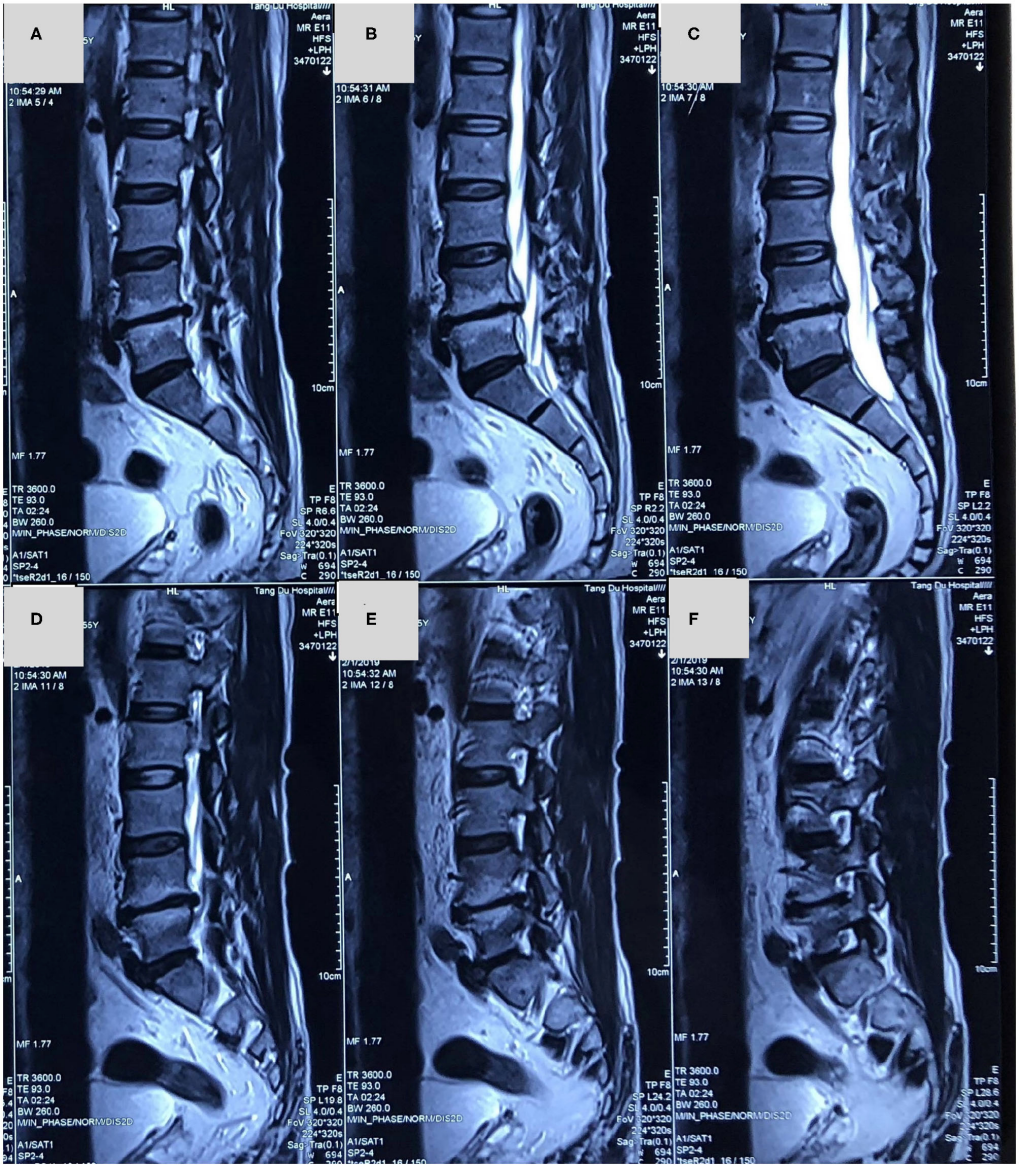
**(A–F)** A magnetic resonance imaging in sagittal slices showed that lumbar disc herniation in L4/5 segment and L4/5 intervertebral disc was in a condition of Modic II type degeneration.

**Figure 3:**
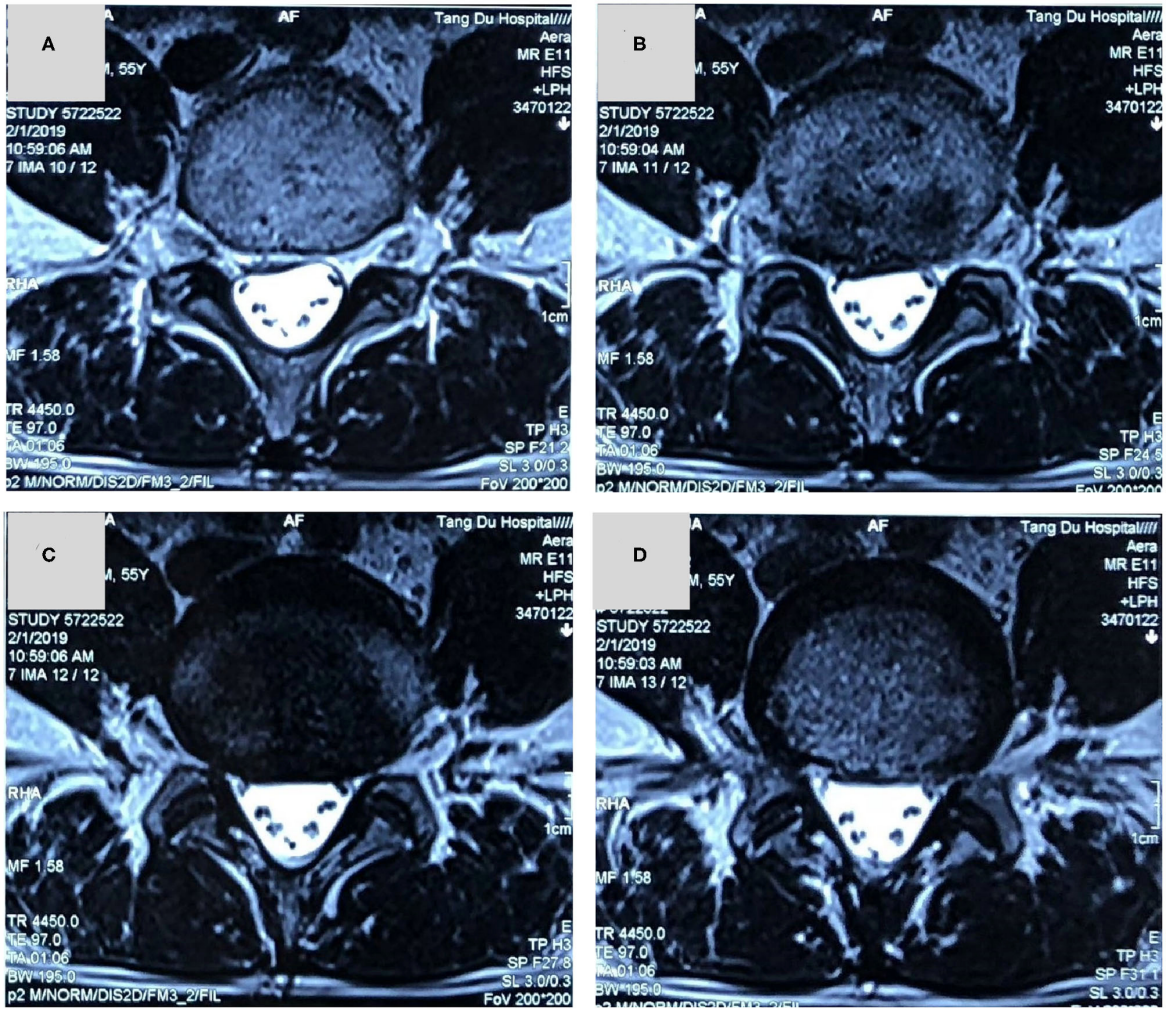
**(A–D)** A magnetic resonance imaging in axial slice showed that a lateral type disc protrusion on the right side in L4/5 and the right lumbar 5th nerve was mildly compressed.

X-ray showed that L4/5 intervertebral height was obviously collapsed (Figure 1). Magnetic resonance imaging (MRI) implied that there was a lateral type disc protrusion on the right side in L4/5 which was in a condition of Modic II type degeneration, mildly pressing the lumbar 5th nerve on the right Figures 2, 3. Thus, the patient was initially diagnosed as sciatica caused by lumbar disc herniation.

### Diagnosis Analysis

#### Supporting Points

1. Previous low back pain (LBP) and history of radiation pain in the right lower extremity, radiation pain in the right lower extremity this time; 2. Physical examination: L4/5 spinous process gap tenderness (+), Lasegue sign left/right = −/+ (60°); 3. Imaging features: intervertebral height collapsed and Modic II type degeneration in L4/5, and a lateral type disc protrusion on the right side in L4/5, pressing the lumbar 5th nerve on the right. Contradiction points: the symptoms of pain in the right lower extremity cannot be relieved by rest or changing posture, and pain is likely continuous knife cutting. In addition, most patients with lumbar disc herniation will have paresthesia, but this patient has no paresthesia on foot.

### Diagnosis and Treatment Process

**Figure 4:**
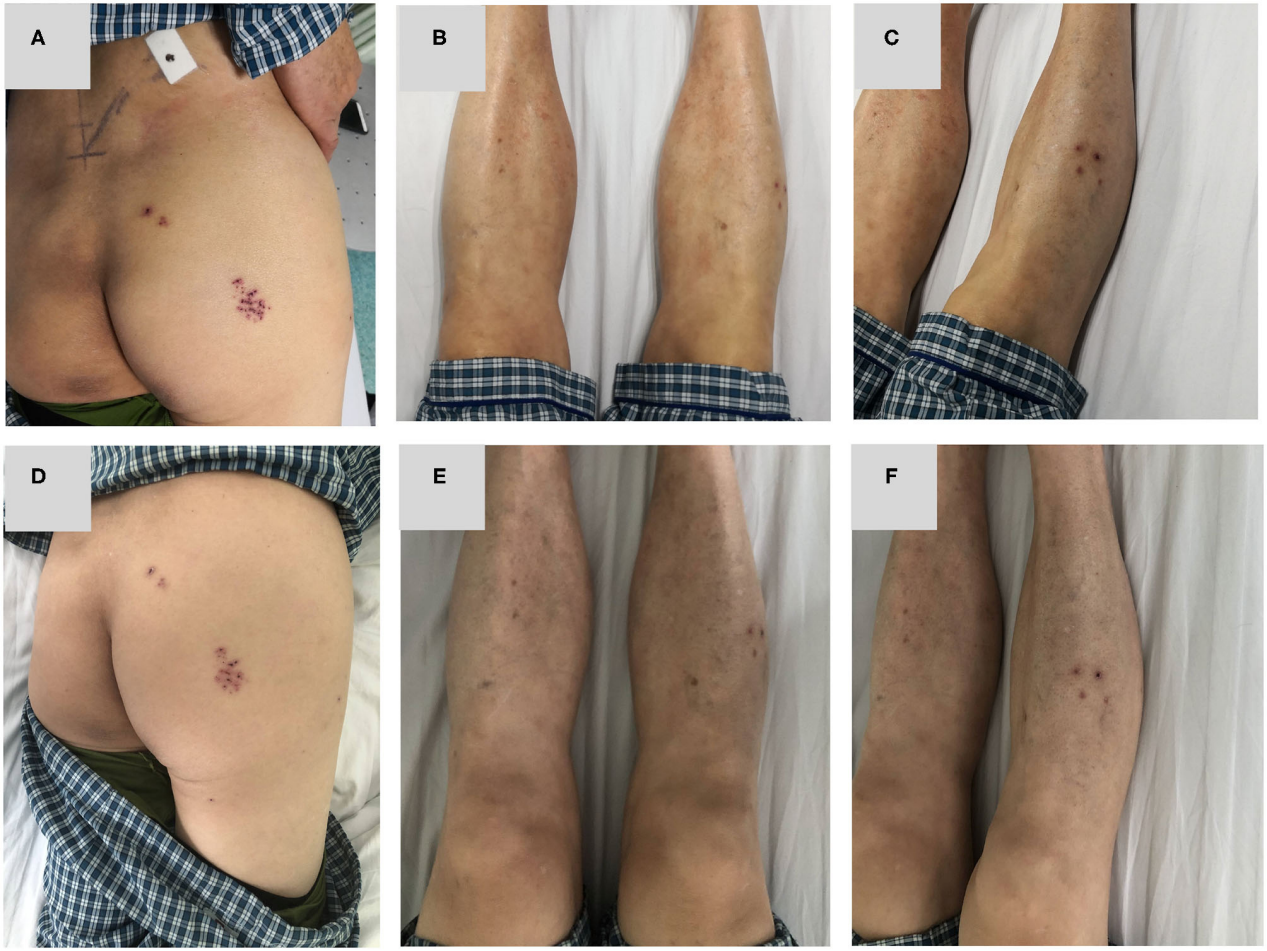
**(A–C)** 3 days after admission, the patient was found to have herpes simplex lesions on the lateral right hip and right calf. **(D–F)** The lesion of the right lower extremity was slightly relieved after taking famciclovir for 3 days.

After 3 days of admission, we examined the body for 3 times successively, and herpes-like skin lesions on the right hip and right calf were found gradually, and local skin was of tenderness (Figures 4A–C). After consultation with a dermatologist, the diagnosis was amended as sciatic herpes zoster in the right lower extremity. Oral administration of famciclovir was recommended and then to observe changes of the symptoms. After 3 days of treatment, the pain in the right lower extremity was of obvious relief, and VAS score was down to 2 points. Physical examination showed that the right lower extremity rash is partially scarred (Figures 4D–F), and the extent of inflammation was subsided significantly. After the treatment, the patients were followed up for one and three months: the pain in the right lower extremity was totally relieved, VAS score was 0, and the rash in the right lower extremity was completely subsided.

## Discussion

The main symptoms of herpes zoster are neuralgia and rash. But the two symptoms appear in chronological order, usually neuralgia appears several days to a week before the rash (5). In this case, the body was examined again and the herpes lesions in the sciatic nerve of the right lower extremity were found after 3 days of admission. Due to the delay in time, the diagnosis is sometimes difficult, especially for patients with a history of lumbar disc herniation and imaging with nerve compression in the corresponding segment. So it is necessary for us to pay attention to the possibility of sciatic HZ about 2 weeks from the onset of lower limb pain.

Several reports have described the differential diagnosis of HZ infection and sciatica originating from the lumbar spine. Sprenger De Rover reported that no abnormal MRI findings in the lumbar spine can rule out that sciatica originated in the lumbar spine (6). It requires careful examination of the medical history, improvement of physical examination, combined with imaging examination to make a clear diagnosis. Otherwise, it is easy to be misdiagnosed, and the surgery that should not be done brings pain and economic burden to the patient (7).

In this patient, there was a history of intermittent LBP and a history of radiation pain in the right lower extremity. This patient's main symptom is radiation pain in the right lower extremity. Physical examination revealed similar signs of lumbar disc herniation: L4/5 spinous process gap tenderness (+), Lasegue sign left/right = −/+. X-ray showed that L4/5 intervertebral height was obviously collapsed. MRI showed there was a lateral type disc protrusion on the right side in L4/5 and the right lumbar 5th nerve was compressed. The initial diagnosis is lumbar disc herniation and is admitted to the hospital for surgical treatment. After admission, the patient's right lower extremity pain was found to be characterized by no relationship with posture and position. The pain was not relieved or aggravated by change of body position. The appearance of the first examination of the spine and limbs was normal. However, after 3 days of admission, the herpes lesions in the sciatic nerve of the right lower extremity were ultimately found. Radiation pain in the right lower extremity was significantly relieved after the application of antiviral drugs. And the right lower extremity sciatic herpes zoster was diagnosed. Continuous antiviral medication was given in accordance with the dermatology consultation. Follow-up in 1 month and 3 months after treatment showed complete relief of the symptoms.

For patients with previous history of lumbar disc herniation, the symptoms of neuralgia caused by herpes zoster are very similar to those of lumbar disc herniation, and it is very easy to be confused by ignoring the inquiry of medical history, physical examination, and differential diagnosis, leading to wrong diagnosis and therapeutic error, or even Performing the operation mistakenly. The characteristic of sciatic herpes zoster is the absence of LBP. In LDH, the patient often complains LBP to some degree (8). It was reported that LBP in LDH may be caused by multiple factors such as discogenic LBP, radicular LBP that is relieved by nerve root decompression, and the LBP due to endplate degeneration (9). Most patients with lumbar disc herniation will have paresthesia, but this patient has no paresthesia on foot. In addition, symptoms are not relieved by rest or change of posture, but are persistent pain. These findings do not support the diagnosis of lumbar disc herniation. This patient did not have back pain and paresthesia on foot on admission this time which did not support the diagnosis of lumbar disc herniation. For pathologic neuralgia caused by herpes zoster, it also has its own unique symptoms and signs: lower limb pain does not relieve or aggravate due to changes in body posture or limb position; After the onset of sciatic nerve transit area, skin lesions such as skin rash gradually appeared. Pain in the lower extremities always show as knife-cutting. Patients often have a history of fatigue, infection and decreased immunity. Collecting medical history comprehensively, repeatedly performing physical examination, and not missing each suspicious symptom will help us to confirm the diagnosis, and to avoid misdiagnosis and missed diagnosis. This is a case report that described a case of suspected lumbar disc herniation with sciatic herpes zoster to reduce the rate of misdiagnosis. The major limitation of the present article is not case series but single case report. The lower extremity sciatic herpes zoster with previous history of LDH is rare clinically. When we have accumulated some cases, we will further analyze the onset characteristics of sciatic herpes zoster in the form of case series.

## Data Availability Statement

The raw data supporting the conclusions of this article will be made available by the authors, without undue reservation.

## Ethics Statement

The studies involving human participants were reviewed and approved by Tangdu hospital ethics committee. The patients/participants provided their written informed consent to participate in this study. Written informed consent was obtained from the individual(s) for the publication of any potentially identifiable images or data included in this article.

## Author Contributions

F-LW, TL, XY, and JQ contributed to study concept and design. YS, L-YB, YY, and CZ provided supervision. F-LW drafted the manuscript. CZ, XY, L-YB, and JQ critically reviewed the manuscript. All authors approved the final version of the manuscript.

## Conflict of Interest

The authors declare that the research was conducted in the absence of any commercial or financial relationships that could be construed as a potential conflict of interest.
